# Is psychological engagement more important than participation? Volunteer activity and subjective cognitive function in older adults

**DOI:** 10.1007/s40520-025-03245-8

**Published:** 2025-11-25

**Authors:** Daijo Shiratsuchi, Yuto Miyake, Ryota Kuratsu, Hiroki Nishi, Manami Fukumori, Shoko Atae, Ryoji Kiyama, Rieko Kosakamoto, Hiromi Tanaka, Hyuma Makizako

**Affiliations:** 1 Department of Physical Therapy, School of Health Sciences, Faculty of Medicine, Kagoshima University, 8-35-1 Sakuragaoka, Kagoshima, 890- 8544 Japan; 2 Graduate School of Health Sciences, Kagoshima University, 8-35-1 Sakuragaoka, Kagoshima, 890-8544 Japan; 3 Kagoshima City Tobu Health Center, 11-1 Yamashitacho, Kagoshima, 892- 8677 Japan

**Keywords:** Social participation, Volunteer activities, Volunteer engagement, Subjective cognitive function

## Abstract

**Background:**

While previous studies have shown inconsistent associations between volunteer participation and cognitive health in later life, less attention has been paid to qualitative aspects of engagement. This study examined whether psychological engagement in volunteer activities is associated with subjective cognitive function in community-dwelling older adults.

**Method:**

This cross-sectional study included 431 community-dwelling older adults (mean age: 78.2 ± 5.7 years; 90.0% female) from self-management exercise class. Volunteer activity status was assessed using a self-reported participation questionnaire. Psychological engagement (Volunteer Engagement; VE) was evaluated using a three-item version of the Utrecht Work Engagement Scale adapted for volunteer settings. Based on VE scores, the participants were categorized into three groups: non-volunteers, low-engagement volunteers, and high-engagement volunteers. Subjective cognitive function was assessed using three self-reported items addressing memory complaints and disorientation, derived from the Kihon Checklist. Logistic regression analyses were conducted to examine the associations between (1) volunteer activity status and (2) VE scores with low cognitive function, adjusting for relevant covariates.

**Results:**

Of 431 participants, 60.1% reported volunteering. There was no significant association between being a volunteer and low cognitive function. In contrast, high-engagement volunteers had significantly lower odds of low cognitive function than non-volunteers (OR = 0.51, 95% CI: 0.27–0.97). No significant differences were observed between low-engagement volunteers and non-volunteers.

**Conclusions:**

These findings suggest that psychological engagement in volunteer activities rather than mere participation may be more closely related to the subjective cognitive health of older adults. Highlighting this engagement may help inform policies and programs for promoting cognitive health.

## Introduction

Dementia is a major public health challenge in aging societies owing to its increasing prevalence and burden on individuals, families, and healthcare systems [1, 2]. The estimated number of people living with dementia worldwide was approximately 57 million in 2019 and is projected to increase to 153 million by 2050, placing a significant burden on healthcare and social systems [1]. Recently, increasing attention has been paid to the importance of early detection and prevention strategies [3, 4]. One promising approach is to focus on factors associated with cognitive function before clinical diagnosis, particularly in older adults who may have low cognitive function without meeting the criteria for dementia [1, 5, 6].

Previous studies reported an association between participation in volunteer activities and higher levels of cognitive function in older adults [7, 8]. Participation in socially productive roles such as volunteering may provide cognitive stimulation, social stimulation, and physical activity, which are considered beneficial for neurological health [8, 9]. However, the findings of previous studies remain inconsistent. Some studies have shown a positive association between volunteering and cognitive outcomes, whereas others have reported null or mixed results [10–12]. These discrepancies may be partly due to different definitions and measurements of volunteer activities, which often focused solely on the presence or absence of participation and may have overlooked the qualitative aspects of the experience. Similar to the distinction between social isolation, an objective state characterized by limited social contacts, and loneliness, a subjective perception of social connectedness [13], volunteer activity can be examined through objective and subjective perspectives. Although previous studies have focused primarily on the objective presence or absence of participation, the subjective psychological aspects, such as engagement and motivation, warrant further investigation.

To address these limitations, recent studies have started to emphasize the importance of the qualitative aspects of volunteer experiences, such as motivation, emotional involvement, and engagement [14, 15]. In particular, the concept of Volunteer Engagement (VE), which is defined as a positive, fulfilling state of mind characterized by vigor, dedication, and absorption in volunteer work, has received increasing attention [16, 17]. VE has been adapted from the psychological construct of work engagement and represents its application in volunteer contexts, offering a more nuanced assessment of psychological involvement than simple participation measures [18]. Despite its potential relevance, to the best of our knowledge, no studies have examined the relationship between VE and cognitive function among community-dwelling older adults.

Therefore, this study aimed to examine the association between VE and cognitive function in community-dwelling older adults. We first explored the association between volunteer activity status and cognitive function by comparing two groups of older adults with and without volunteer activities. First, we explored the association between volunteer activity status and cognitive function by comparing volunteers and non-volunteers. We then classified the participants into three groups based on their levels of activity and psychological engagement: non-volunteers, low-engagement volunteers, and high-engagement volunteers, and examined their associations with cognitive function.

## Methods

### Participants

This cross-sectional observational study was conducted between September and October, 2024 in Kagoshima City, Japan. Participants were recruited from a community-based self-management exercise class known as the Yokayoka Genki Club, which is primarily intended for adults aged ≥ 65 years. The Yokayoka Genki Club is a community-based group activity held approximately once a week at local public halls and meeting places. It is composed primarily of five or more older adults and includes light exercise and periodic instructions from physical therapists and other professionals. In total, 454 individuals participated in the survey. After excluding those aged < 65 years (*n* = 11), those with a history of stroke (*n* = 11), or Parkinson’s disease (*n* = 1), the final sample for analysis consisted of 431 participants (mean age: 78.2 ± 5.7 years; female: 90.0%). All participants were informed of the purpose and procedures of the study and provided written informed consent before participation. The study protocol was approved by the Ethics Committee on Epidemiological and Related Studies at Sakuragaoka Campus, Kagoshima University (approval number: 230302).

### Volunteer activity and engagement

Volunteer activity status was assessed using a self-administered questionnaire that included the question, ‘Do you engage in charity or volunteer activities?’ [19] Participants who responded “yes” were classified as volunteers, and those who responded “no” were classified as non-volunteers.

VE scores were assessed among participants who reported engaging in volunteer activities using a three-item version of the Utrecht Work Engagement Scale (UWES-3) [20], with the word “work” replaced by “voluntary work” to reflect the context [16]. The items were: (1) “At my voluntary work, I feel bursting with energy” (vigor), (2) “I am enthusiastic about my voluntary work” (dedication), and (3) “I am immersed in my voluntary work” (absorption). Responses were rated on a 7-point Likert scale ranging from 0 (never) to 6 (always), and the mean score of the three items was calculated for each participant. Based on the distribution of VE scores, the participants were divided into tertiles. Those in the top tertile were classified as high-engagement volunteers, and those in the lower two tertiles were classified as low-engagement volunteers. Combined with the non-volunteers, the final sample was divided into three groups for analysis: non-volunteers, low-engagement volunteers, and high-engagement volunteers. The UWES-3 was selected because it is a brief and validated measure widely used to assess psychological engagement through the core dimensions of vigor, dedication, and absorption [20]. This scale was deemed most practical for capturing core engagement constructs in this community-based study, while minimizing participant burden compared to longer or more complex instruments.

### Cognitive function

Subjective cognitive function was assessed using three items from the cognitive function domain of the Kihon Checklist developed by the Japanese Ministry of Health, Labor and Welfare [21]. The items were: (1) “Do your family or your friends point out your memory loss? e.g., “You ask the same question over and over again.” (risk response: yes), (2) “Do you make a call by looking up phone numbers?” (risk response: no), and (3) “Do you find yourself not knowing today’s date?” (risk response: yes) [22]. Each item was answered in binary format (“yes” or “no”). Participants who provided at least one risk response were classified as having low cognitive function, following a screening approach used in previous KCL-based studies for identifying subjective cognitive problems [23]. This definition is supported by previous research showing that endorsing at least one item in the cognitive domain of the Kihon Checklist is associated with an increased risk of developing dementia [22].

### Other variables

Based on previous studies, the following variables were assessed [24, 25]: age, sex, educational level, body mass index (BMI), presence of chronic diseases (hypertension, hyperlipidemia, and diabetes mellitus), living status (living alone), polypharmacy status, and depressive symptoms. The measurements and categorizations are described below. Most variables were obtained through a tablet-based, self-administered questionnaire, with the research staff available to provide explanations or assistance as needed. BMIs were calculated from the participants’ measured heights and weights.

Educational level was assessed by asking participants how many years of formal education they had completed. Responses were categorized into four groups (< 6 years, 6–9 years, 10–12 years, and ≥ 13 years), and then dichotomized as ≤ 12 years or ≥ 13 years for analysis [26].

Polypharmacy was defined as the regular use of six or more medications based on the Guidelines for Medical Treatment and Its Safety in the Elderly 2015 in Japan [27], which indicates an increased risk of adverse drug events with this threshold.

Depressive symptoms were assessed using the five-item version of the Geriatric Depression Scale (GDS-5) [28], a brief screening tool commonly used in older adults. Each item was answered with “yes” or “no,” and total scores ranged from 0 to 5, with higher scores indicating more severe depressive symptoms. Participants with a total score of less than 2 were classified as not having depressive symptoms, whereas those with a score of 2 or higher were classified as having depressive symptoms [29].

### Statistical analyses

Descriptive statistics were calculated for all variables. Continuous variables are presented as means and standard deviations, and categorical variables are presented as frequencies and percentages. To compare participant characteristics by volunteer activity status (volunteers vs. non-volunteers), unpaired t-tests were used for continuous variables, and the chi-squared test was used for categorical variables. To compare participant characteristics by volunteer activity and engagement level (non-volunteers, low-engagement volunteers, and high-engagement volunteers), one-way analysis of variance (ANOVA) was used for continuous variables, and the chi-squared test was used for categorical variables. When significant group differences were observed, Bonferroni-adjusted post hoc comparisons were conducted.

Two sets of logistic regression analyses were conducted to examine the associations between (1) volunteer activity status and (2) volunteer activity and engagement levels with low cognitive function. Two models were constructed for each analysis: a crude model and an adjusted model. In the first analysis, the volunteer activity status (volunteer vs. non-volunteer) was used as an independent variable. In the second analysis, a three-category variable representing volunteer activity and engagement levels (non-volunteers, low-engagement volunteers, and high-engagement volunteers) was used, with non-volunteers as the reference. The adjusted models included the following covariates: age, sex, educational level (≤ 12 years), polypharmacy (≥ 6 medications), and depressive symptoms (GDS-5 score ≥ 2). All statistical analyses were performed using SPSS Statistics version 30.0 (IBM Corp., Armonk, NY, USA). Statistical significance was defined as a two-sided p-value of < 0.05.

## Results

### Participant characteristics by volunteer activity status

The demographic characteristics of the participants, stratified by volunteer activity status, are presented in Table 1. Of the 431 participants, 259 (60.1%) were classified as volunteers and 172 (39.9%) as non-volunteers. Overall, 142 participants (32.9%) had low cognitive function. Compared to non-volunteers, volunteers were significantly younger (77.4 ± 5.5 vs. 79.4 ± 5.9 years; *P* < .001) and had a lower proportion of females (86.9% vs. 94.8%; *P* = .007). Additionally, volunteers had a significantly lower proportion of individuals with ≤ 12 years of education (*P* = .012), a lower prevalence of living alone (*P* = .041), and fewer participants with depressive symptoms (*P* < .001).

**Table 1:** Demographic characteristics of participants by volunteer activities

Variables	Overall (*n* = 431)	Non-volunteer group (*n* = 172)	Volunteer group (*n* = 259)	*P*-value
Age, y, mean ± SD	78.2 ± 5.7	79.4 ± 5.9	77.4 ± 5.5	< 0.001
Sex, female (%)	388 (90.0)	163 (94.8)	225 (86.9)	0.007
Education ≤ 12 years, n (%)	337 (78.2)	145 (84.3)	192 (74.1)	0.012
Body mass index, kg/m^2^, mean ± SD	22.9 ± 3.2	23.1 ± 3.5	22.8 ± 3.0	0.385
Chronic disease, n (%)				
Hypertension	170 (39.4)	77 (44.8)	93 (35.9)	0.065
Hyperlipidemia	90 (20.9)	35 (20.3)	55 (21.2)	0.824
Diabetes mellitus	42 (9.7)	19 (11.0)	23 (8.9)	0.458
Living alone, n (%)	141 (32.7)	66 (38.4)	75 (29.0)	0.041
Polypharmacy (≥ 6 meds), n (%)	38 (8.8)	15 (8.7)	23 (8.9)	0.954
GDS-5 score ≥ 2, n (%)	71 (16.5)	42 (24.4)	29 (11.2)	< 0.001

GDS, Geriatric Depression Scale

### Participant characteristics by VE scores

The demographic characteristics of the participants, categorized by VE scores, are summarized in Table 2. Significant group differences were observed for age (*P* < .001), with non-volunteers (a) being older than low-engagement volunteers (b) (a > b). The non-volunteer group also had a higher proportion of females (*P* = .028, non-volunteers > low-engagement volunteers), more individuals with ≤ 12 years of education (*P* = .034, a > b), and a greater prevalence of living alone (*P* = .026, non-volunteers > low-engagement volunteers). Regarding depressive symptoms (GDS-5 scores ≥ 2), non-volunteers showed a higher prevalence than both low-engagement and high-engagement volunteers (*P* < .001, non-volunteers > low-engagement volunteers, non-volunteers > high-engagement volunteers). No significant differences were observed between the low-engagement and high-engagement volunteers regarding any demographic characteristics.

**Table 2:** Comparison of demographics by volunteer engagement scores

Variables	Non-volunteer group^a^ (*n* = 172)	Low-engagement volunteer group^b^ (*n* = 177)	High-engagement volunteer group^c^ (*n* = 82)	*P*-value	Post hoc
Age, y, mean ± SD	79.4 ± 5.9	77.0 ± 5.8	78.3 ± 4.8	< 0.001	a > b
Sex, female (%)	163 (94.8)	154 (87.0)	71 (86.6)	0.028	a > b
Education ≤ 12 years, n (%)	145 (84.3)	129 (72.9)	63 (76.8)	0.034	a > b
Body mass index, kg/m^2^, mean ± SD	23.1 ± 3.5	22.6 ± 2.9	23.3 ± 3.3	0.143	
Chronic disease, n (%)					
Hypertension	77 (44.8)	64 (36.2)	29 (35.4)	0.182	
Hyperlipidemia	35 (20.3)	39 (22.0)	16 (19.5)	0.876	
Diabetes mellitus	19 (11.0)	15 (8.5)	8 (9.8)	0.720	
Living alone, n (%)	66 (38.4)	45 (25.4)	30 (36.6)	0.026	a > b
Polypharmacy (≥ 6 meds), n (%)	15 (8.7)	12 (6.8)	11 (13.4)	0.215	
GDS-5 score ≥ 2, n (%)	42 (24.4)	24 (13.6)	5 (6.1)	< 0.001	a > b; a > c

GDS, Geriatric Depression Scale

### Low cognitive function by VE scores

**Fig. 1:**
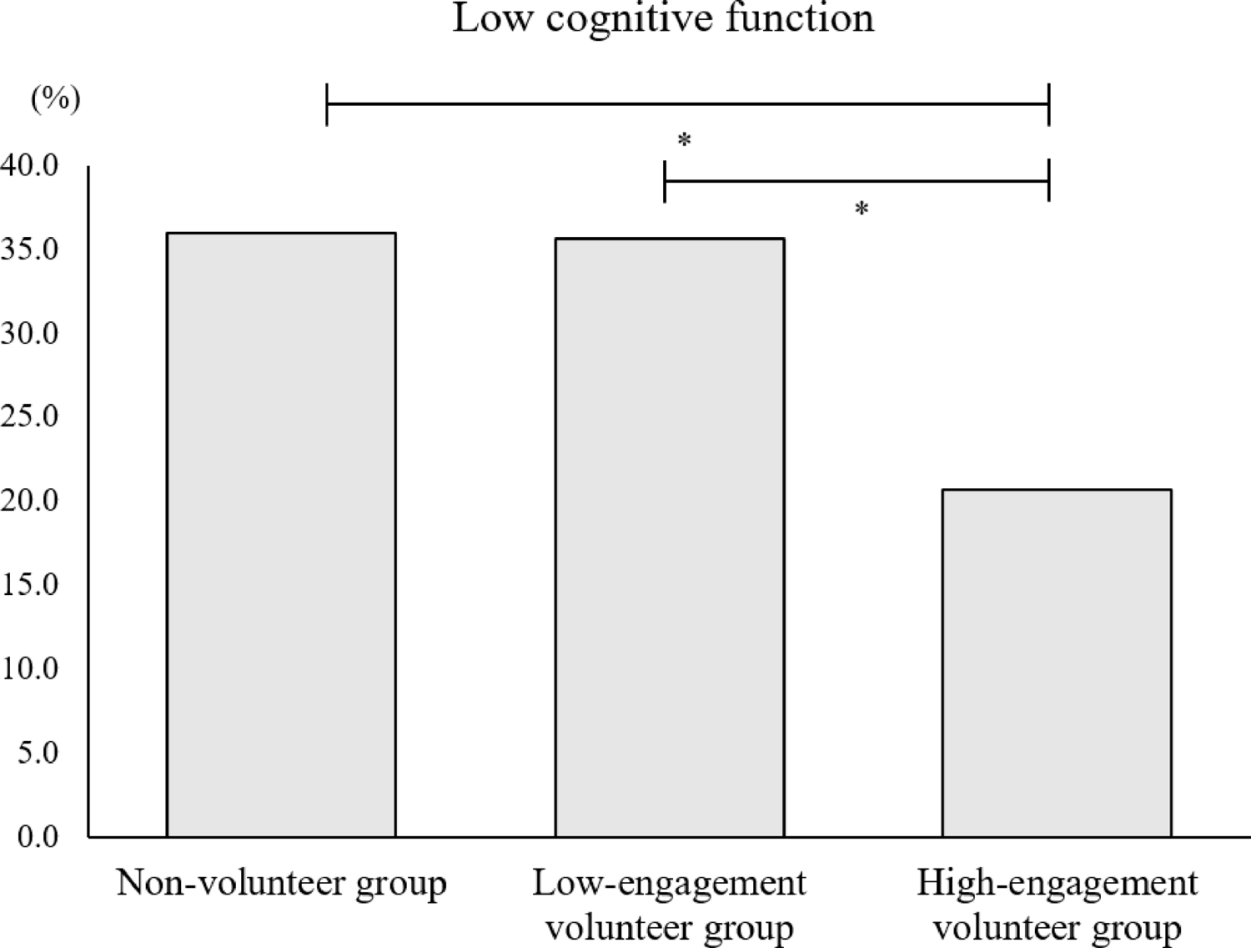
Proportion of participants with low cognitive function by Volunteer Engagement scores. The proportions of participants classified as having low cognitive function in each group were as follows: non-volunteers (36.0%), low-engagement volunteers (35.6%), and high-engagement volunteers (20.7%). Significant group differences were observed (*P* = .033), with highly engaged volunteers showing a significantly lower proportion than the other two groups (*P < 0.05)

The proportion of participants with low cognitive function categorized by VE scores is shown in Fig. 1. A significant difference was observed between the groups (*P* = .033). The prevalence of low cognitive function was 36.0% in non-volunteers, 35.6% in low-engagement volunteers, and 20.7% in high-engagement volunteers. Both the non-volunteer and low-engagement volunteer groups had significantly higher proportions than the high-engagement group (non-volunteers > high-engagement volunteers, low-engagement volunteers > high-engagement volunteers), whereas there was no significant difference between the non-volunteer and low-engagement groups.

### Association between volunteer activities and low cognitive function

Table 3 presents the association between volunteering and low cognitive function. Neither the crude model (odds ratio [OR]: 0.79, 95% confidence interval [CI]: 0.53–1.19; *P* = .265) nor the adjusted model (OR: 0.88, 95% CI: 0.57–1.35; *P* = .551) showed a statistically significant association between volunteering and low cognitive function.

**Table 3:** Association between volunteer activities and low cognitive function

Independent variable	Crude		Adjusted model	
	OR (95% CI)	P-value	OR (95% CI)	P-value
Non-volunteers	Reference		Reference	
Volunteers	0.79 (0.53–1.19)	0.265	0.88 (0.57–1.35)	0.551

Adjusted model: Age, sex, education (≤ 12 years), polypharmacy (≥ 6 medications), GDS-5 (score ≥ 2)

Abbreviations: 95% CI, 95% confidence interval; OR, odds ratio

### Association between VE scores and low cognitive function

Table 4 presents the association between VE scores and low cognitive function. High-engagement volunteers had significantly lower odds of low cognitive function compared to non-volunteers in both the crude model (OR: 0.46, 95% CI: 0.25–0.86; *P* = .015) and the adjusted model (OR: 0.51, 95% CI: 0.27–0.97; *P* = .040).

**Table 4:** Association between volunteer engagement scores and low cognitive function

Independent variable	Crude		Adjusted model	
	OR (95% CI)	P-value	OR (95% CI)	P-value
Non-volunteers	Reference		Reference	
Low-engagement volunteers	0.98 (0.63–1.52)	0.930	1.09 (0.69–1.72)	0.720
High-engagement volunteers	0.46 (0.25–0.86)	0.015	0.51 (0.27–0.97)	0.040

Adjusted model: Age, sex, education (≤ 12 years), polypharmacy (≥ 6 medications), GDS-5 (score ≥ 2)

Abbreviations: 95% CI, 95% confidence interval; OR, odds ratio

Low-engagement volunteers showed no significant association with low cognitive function compared to non-volunteers in either the crude model (OR: 0.98, 95% CI: 0.63–1.52; *P* = .930) or the adjusted model (OR: 1.09, 95% CI: 0.69–1.72; *P* = .720).

## Discussion

This study examined the association between volunteer-related factors and subjective cognitive function in community-dwelling older adults. While no significant association was found between volunteering and low cognitive function, a significant association was observed when considering VE scores, aligning with our initial hypothesis. Specifically, older adults with high VE scores had significantly lower odds of being classified as having low cognitive function than non-volunteers, even after adjusting for relevant covariates. In contrast, low-engagement volunteers did not differ significantly from non-volunteers. These findings suggest that a higher level of psychological engagement in volunteer activities, rather than mere participation, may be more closely associated with better cognitive health in older adults.

Previous studies have reported mixed findings regarding the association between volunteer participation and cognitive function in older adults [6]. A recent comprehensive review by Sommerlad et al. [6]. summarized observational evidence suggesting that greater social participation in mid- and late-life is associated with a 30–50% lower risk of developing dementia. However, as the authors also noted, there is considerable heterogeneity in how volunteer participation is measured. We believe that many previous studies may have overlooked important qualitative aspects such as motivation and psychological engagement. Our finding that volunteering was not significantly associated with cognitive function is consistent with those of previous studies that reported null or inconsistent results [30–32]. By incorporating the concept of VE scores, which reflects psychological involvement in volunteer work, our study provides novel insights into the potential relevance of the qualitative dimensions of social participation. To the best of our knowledge, research examining the association between VE scores and subjective cognitive function is limited, suggesting that the present findings offer a novel perspective. Thus, these insights may help guide the development of community programs and policies that increase volunteer participation as well as foster meaningful and psychologically engaging experiences for older adults.

Several mechanisms may explain the observed association between high VE scores and the lower likelihood of low subjective cognitive function. Guiney and Machado have suggested that volunteering may promote cognitive health in older adults by increasing physical, social, and cognitive activities, each of which is independently associated with better cognitive outcomes [33]. In cultural contexts such as Japan, where community-based volunteer roles may be shaped by social expectations such as those found in neighborhood associations, some individuals may participate out of obligation rather than intrinsic motivation, resulting in lower psychological engagement [34, 35]. However, a strong sense of communal belonging and identification can collectively enhance satisfaction and commitment to volunteering, as shown in collectivist settings [36]. Moreover, cultural values may influence the perception and reporting of memory difficulties. For instance, older adults in collectivist societies, such as Japan, may be less likely to openly acknowledge cognitive concerns owing to stigma or fear regarding burdening others [37]. Such tendencies may result in lower reporting of subjective cognitive complaints. In our study, 142 individuals (32.9%) were classified as having low cognitive function, which is within the internationally observed range of 6.1%–52.7% reported by Röhr et al. [38], indicating that substantial underreporting was unlikely.

High VE scores may reflect deeper psychological involvement and greater intensity of engagement in volunteer activities, potentially leading to increased opportunities for learning, problem solving, and meaningful social interaction. Additionally, engagement in cognitively stimulating social roles may support the maintenance of cognitive function [39] and may be associated with fewer depressive symptoms [40], which are, in turn, linked to cognitive health in later life. Together, these findings suggest that VE scores may reflect not only the level of participation but also the degree of intrinsic motivation and psychological engagement in volunteer activities. However, further longitudinal and experimental studies are required to clarify the directionality and underlying mechanisms of these associations.

This study had several limitations. First, owing to its cross-sectional design, causal relationships cannot be inferred. It is possible that individuals with better preserved cognitive function are more likely to engage in and derive meaning from volunteer activities rather than the reverse. Second, cognitive functions were assessed using a brief self-reported measuring tool, which may have been influenced by factors such as mood, personality traits, and insight. Objective neuropsychological testing was not performed. Additionally, we did not collect information on the specific content, frequency, or duration of volunteer activities, which may have influenced both engagement levels and cognitive outcomes. Third, the sample mainly comprised women and individuals already participating in a community-based self-management exercise class, potentially limiting the generalizability of our findings, as the participants tended to be socially connected. Finally, although we adjusted for key demographic and health-related covariates, the potential for residual confounding factors remains unchanged.

## Conclusion

The findings of this study showed that high VE scores were significantly associated with a lower likelihood of being classified as having low subjective cognitive function, whereas the mere presence or absence of volunteer activities was not. This finding suggests that psychological engagement in volunteer activities, rather than participation only, may be more closely related to cognitive health in older adults. These results highlight the importance of incorporating the qualitative aspects of engagement into the design and promotion of volunteer opportunities for older adults. Further longitudinal and interventional studies are needed to clarify these causal relationships.

## Data Availability

The data used in this study cannot be publicly shared due to privacy concerns and ethical restrictions. The data can be obtained from the corresponding authors upon reasonable request.
